# Multi-objective optimization of covering parameters for cotton/spandex core-spun yarn using grey relational analysis in conjunction with the Taguchi technique

**DOI:** 10.1038/s41598-025-29994-0

**Published:** 2025-12-16

**Authors:** Alsaid Ahmed Almetwally

**Affiliations:** Textile Engineering Department, Textile Research and Technology Institute, National Research Center, Dokki, Cairo, Egypt

**Keywords:** Multi-objective optimization, Cotton, Spandex, Core-spun yarn, Grey relational analysis, Taguchi technique, Process parameters, Engineering, Materials science, Mathematics and computing

## Abstract

The growing need for high-performance stretchable fabrics led scientists to innovate a new spinning technique, especially for manufacturing cotton/spandex core-spun yarns. This type of yarn is spun by using spandex monofilament or multifilament as a core, which is surrounded by a sheath of staple cotton fibers. The key covering process parameters include spindle speed, delivery roller speed, spandex drafting ratio, spandex linear density, and tension level, which simultaneously influence the core-spun yarn characteristics such as tensile properties, hairiness index, imperfection index, and fabric aesthetic and performance properties. Fine-tuning these multiple covering parameters achieves optimal performance of these types of yarns. This paper aimed at employing multi-objective optimization for the covering parameters of cotton/spandex composite yarn to maximize the yarn tensile properties and minimize both hairiness and imperfection indices using the robust Taguchi technique in conjunction with the grey relational analysis. A full factorial design composed of three factors, namely spandex monofilament drafting ratio, linear density, and core-spun yarn twist multiplier, with five, four, and two levels, was conducted. Average values of the grey relational grades of all combinations were estimated, and its highest value refers to the optimal combinations of the controllable factors, which yield the best performance of cotton/spandex core-spun yarn. This study revealed that core-spun yarn with a 4.2 twist multiplier, a 44 dtex linear density of spandex monofilament, and a 4.4 drafting ratio of spandex yielded the optimal yarn performance characteristics. This study provides a methodological breakthrough with beneficial ramifications for the textile industry seeking a multi-objective optimization of core-spun yarn manufacturing parameters.

## Introduction

A textile structure made up of a sheath and a core is referred to as a core-spun yarn. The core is typically made up of a synthetic, rigid, or elastic monofilament or multifilament. The sheath, which surrounds the core, is commonly composed of synthetic or natural staple fibers like wool, polyester, cotton, etc^1–3^. The core generally imparts the functional and mechanical characteristics of this kind of spun yarn. At the same time, cotton fibers are widely utilized as a sheath due to their superior absorbency, soft hand feel, comfort, and other exceptional attributes^4^.

Various spinning techniques can be employed to spin core-spun yarns; for example, air-jet spinning, rotor spinning, friction spinning, and ring spinning are the most common techniques^5–7^. This type of spun yarn is primarily manufactured to ameliorate the aesthetics, durability, strength, and performance characteristics of the woven fabrics^8,9^.

The important aspects of the core-spinning process include the type of core material, type of sheath fibers, linear density of the core filament, yarn twist multiplier, drafting ratio of the core filament, and core-spun yarn count, etc. Numerous research works have examined how the spinning variables influence the mechanical and physical characteristics of core-spun yarns. Samah et al. examined the impact of core-spun yarn structure and its linear density, including elastic core/T400, elastic core/spandex, dual-core, and tri-core structures, on their physical and mechanical properties. Their findings indicated that both yarn structures and linear density, along with their interaction, significantly influenced the physical and mechanical characteristics of the core-spun yarns except for the hairiness index^10^. Sevim and Demet produced core-spun yarns composed of PBT and X55 filaments with a spandex filament as a core, which were wrapped with different sheath fibers, such as cotton, viscose, and cotton/Tencel. The effects of various fiber and filament types in the sheath and core along with the yarn fineness on the mechanical properties of spun yarns and characteristics of the woven fabrics were statistically examined. Their findings revealed that dual-core yarns wrapped with cotton fibers as the sheath exhibited high yarn irregularity, imperfection values, and hairiness, along with lower tensile characteristics. On the contrary, yarns wrapped with a Tencel/cotton fiber blend disclosed improved values for the above yarn properties. Similarly, viscose sheath fibers yielded results comparable to the dual-core yarns with blended fibers. They also unexpectedly found that dual-core yarns with Tencel and viscose sheath fibers exhibited higher tenacity compared to those produced with blended sheath fibers. The tensile strength and elongation at break of dual-core yarns were directly reflected in the tensile properties of the corresponding woven fabrics. Additionally, fabrics woven from dual-core yarns containing spandex and X55 filaments showed superior tensile characteristics compared to those produced from PBT and spandex core filaments^11^. In a similar work, Rashel et al. produced two types of core-spun yarns, namely single-core and dual-core-spun yarns. Cotton fibers make up the sheath in both yarns, while spandex monofilament and polyester multifilament constitute the core. Their findings revealed that dual-core spun yarns demonstrated lower hairiness and imperfection indices and irregularity compared to single-core spun yarns. Furthermore, it was disclosed that the dual-core spun has higher bundle stress than single-core spun yarns^12^.

Cheng and his fellow accounted for and analyzed the high-frequency defects occurring in cotton/spandex core-spun yarn during its manufacturing on ring spinning machines. Their analysis revealed that cotton/spandex core-spun yarns primarily had common defects, such as uneven elasticity, irregularity, hairiness, partial yarn, bare yarn, and hollow core, during the manufacturing process. Partial defect was found to have the highest frequency among them, followed by yarn irregularity. They also emphasized that the quality of cotton/spandex yarn and the occurrence of associated defects can be substantially improved by adopting effective strategies in three key areas: strengthening equipment management, adapting optimization techniques of process parameters, and stringent personnel operation management^13^. Ikramul and Ahmed compared cotton/spandex core-spun yarns of different counts produced by traditional and compact spinning systems in terms of their physical and mechanical properties. Their results revealed that compact core-spun yarns exhibited superior properties compared to their counterparts spun on a traditional system. The superiority of core-compact spun yarns includes a lower hairiness index, reduced irregularity and imperfection values, especially seed-coat neps, and higher tensile properties for all yarn counts. They also concluded that pneumatic compact spinning is the most suitable manufacturing system for producing cotton/spandex core-spun yarns with a unique structure and enhanced properties^14^. Alsaid Ahmed Almetwally and co-authors, forecasted the tensile characteristics of cotton/spandex core-spun yarns using Artificial Neural Networks (ANN) and compared the results with those obtained from a linear regression model. The findings demonstrated that the ANN outperformed the regression model, with lower root mean square error and mean bias error, as well as a higher coefficient of determination value^15^.

Finally, the impact of cotton/spandex core-spun yarn parameters on the stretchable woven fabric properties was also addressed in various research^16–26^. To the best of the author’s knowledge, the optimization of covering process parameters of the core-spun yarns, particularly cotton/spandex core-spun yarns, has not been addressed in previous research works. Therefore, the overarching aim of this study is the multi-objective optimization of cotton/spandex core-spun yarn parameters. The twist multiplier of the core-spun yarn, the linear density and drafting ratio of its spandex core monofilament were considered. Throughout this study, grey relational analysis in conjunction with the Taguchi robust technique was employed to maximize the breaking load, elongation at break, and breaking work of the cotton/spandex core-spun yarn, while minimizing its imperfection and hairiness indices.

## Experimental work

### Materials

Throughout this study, forty cotton/spandex yarn samples with a net count of 30/1 Ne (19.68 tex) were produced on the core spinning machine of model Sinzer 513 spinning machine under varying processing conditions, including two twist multipliers of the yarn, four linear densities of spandex monofilament, and five spandex drafting ratios. The cotton fibers, which were used as a sheath and wrapped around the core of spandex monofilament, were of Egyptian cotton type Giza 86, whose characteristics are presented in Table 1. Similarly, the characteristics of the spandex monofilament, which was used as a core, were also summarized in Table 2.

**Table 1:** Characteristics of Egyptian cotton Giza 86 used as a sheath.

Color	White
Upper Half Mean Length, (mm)	34
Uniformity Index	88.4
Strength, (g/tex)	43
Elongation, (%)	6.5
Fiber perimeter, (μ)	47.2
Total reducing sugar, (%)	0.14
Brightness, (Rd %)	76.4
Yellowness, (+ b)	9.4
Micronaire value	4.2
Maturity ratio	0.94

**Table 2:** Characteristics of Spandex monofilament used as a core.

	Spandex monofilament’s linear density, denier (dtex)			
	19.8 (22)	29.7 (33)	39.6 (44)	70.2 (78)
Luster	Clear	Clear	Clear	Clear
Tenacity, g/tex	13.9 ± 1.35	13.5 ± 1.35	12.6 ± 1.35	11.25 ± 1.35
Breaking elongation, %	650 ± 50	650 ± 50	640 ± 50	700 ± 70

It should be noted that the 30/1 Ne yarn count is considered a medium count. Therefore, it is commonly used in different textile products, including men’s shirts, women’s skirts, and underwear for both genders. Accordingly, it was selected to be investigated throughout this study.

### Core spinning machine and influencing parameters

A covering process is generally used to produce core-spun yarns, or specifically to cover the core (spandex monofilament in this study) with drawn cotton staple fibers in the traditional ring spinning machine. To accomplish this task, the spinning machine is fitted with a device to feed spandex filament positively, which also controls its drafting ratio.

**Fig. 1:**
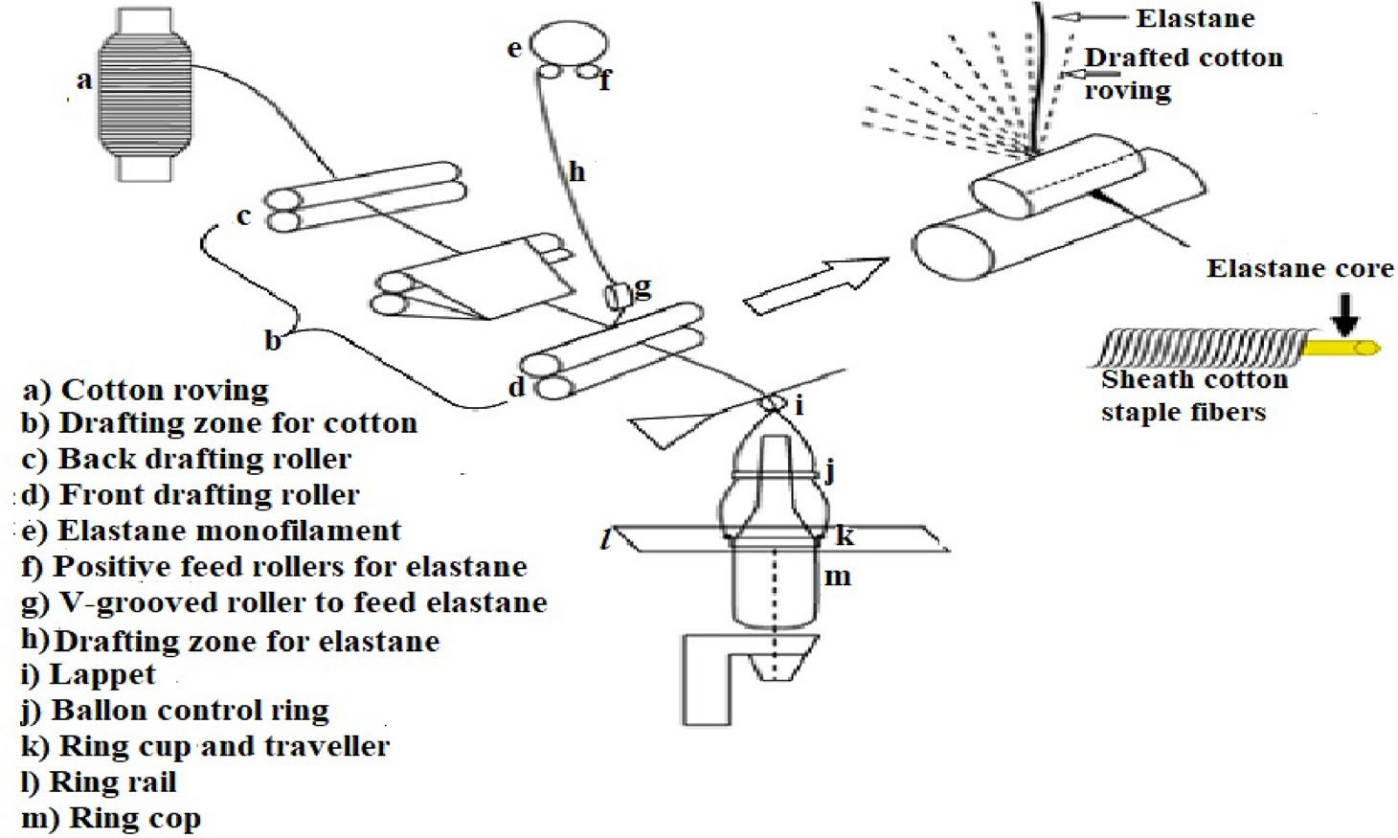
Passage of cotton fibers and spandex monofilament through Zinser 513 spinning machine for spinning cotton/spandex core-spun yarn^14^.

The cotton/spandex core-spun yarns used in this study were produced on a Zinser 513 spinning machine, which is specifically designed for manufacturing core-sheath structured yarns. This machine feeds the spandex monofilament at the center of the front rollers, which is subsequently wrapped by the drawn staple cotton fibers^14^. All cotton/spandex core-spun yarn samples investigated in this study have the same count of 30/1 Ne and were spun from drafting a cotton roving sliver of count 0.9 Ne, with a total drafting ratio of 33. Figure 1 demonstrates the passage of cotton fibers and spandex monofilament through the Zinser spinning machine during the production of the examined core-spun yarns.

The Egyptian raw cotton of type Giza 86 underwent several successive processing steps, summarized as follows: Initially, the cotton fibers were opened and cleaned using a Trützschler opening and cleaning line, which removed over 70% of the trash and impurities. The processed cotton exited this stage in the form of a mat, which was then fed into a carding machine of the model (FA203 C-China) for further cleaning. In this stage, additional impurities, neps, and short fibers were removed, and the fibers were individualized. These fibers were subsequently formed into a web and delivered from the carding machine as a continuous cylindrical carded sliver with a linear density of 4 K tex. Thereafter, to improve fiber parallelization and enhance yarn uniformity, the carded sliver underwent two successive drawing stages—breaker and finisher drawing—using Toyoda DX-8 and Rieter RSB D-22 machines, respectively. The drawn sliver was further processed on a Toyoda FL-16 roving machine to convert it into roving of count 0.96 Ne. This roving was then fed into a Zinser 513 spinning machine, equipped with a special attachment to accurately feed the core yarn, spandex monofilament, at a controlled and stable rate. The principle of manufacturing cotton/spandex core-spun yarns is depicted in Fig. 1.

The key parameters of this machine that significantly influence the physical and mechanical properties of the produced yarns include the type of the cotton fibers, yarn linear density, yarn twist multiplier, spandex monofilament draft ratio, drafting system engineering, and spandex monofilament linear density.

In this study, the investigation was confined to the following controllable factors: the yarn twist multiplier, drafting ratio, and linear density of the spandex monofilament.

The twist multiplier (α_e_) of the core-spun yarn was controlled by varying the spindle speed, V_m_, and the delivery roller speed, V_d_, in the figure, and it can be calculated by the following equations:

1 $$Turns/m = \frac{{Spindle.speed,V_{m} \left( {rpm} \right)}}{{Fron.rokller.speed,V_{d} \left( {m/\min } \right)}}$$

2 $$Turns/inch = \alpha_{e} \times \sqrt {N_{e} }$$

The drafting ratios applied to the spandex monofilament can be calculated by dividing the front roller speed (V_d_) by the speed of feed rollers for spandex (V_f_), as illustrated in the following formula:

3 $$Drafting.ratio\left( {DR} \right) = \frac{{Fron.roller.speed,V_{d} \left( {m/\min } \right)}}{{Spandex.feed.roller.speed,V_{f} \left( {m/\min } \right)}}$$

In this study, each spandex monofilament linear density was subjected to five different drafting ratios, namely 2.4, 2.8, 3.3, 4, and 4.4, respectively. As a result, the amount of spandex in each of the forty produced yarns varied significantly based on both spandex drafting ratio and linear density. Therefore, the mechanical and physical characteristics of the produced yarns will also differ substantially.

The following equation can be utilized to compute the amount of spandex incorporated in the cotton/spandex yarn:

4 $$P\% = \frac{{Spandex.linear.density\left( {tex} \right)}}{{Drafting.ratio.of.spandex\left( \% \right) \times Yarn.count.with.spandex\left( {tex} \right)}} \times 100$$

### Experimental design

To examine the influence of chosen core-spinning parameters on the properties of cotton/spandex core-spun yarn, a full factorial design was employed. In this study, three controllable factors were considered: twist multiplier of the cotton/spandex core spun yarn with two levels, linear density of spandex monofilament with four levels, and drafting ratio of spandex monofilament with five levels. Accordingly, a full and mixed 2 × 4 × 5 factorial design was conducted, resulting in a total of 40 experiments run. The effects of controllable factors on the cotton/spandex core-spun yarns’ characteristics, including breaking load, breaking elongation, breaking work, hairiness index, and total imperfection index, were systematically investigated. The chosen control factors and their levels are introduced in Table 3. The designed combinations of controllable parameters and their corresponding cotton/spandex core-spun yarn properties were tabulated in Table 4.

**Table 3:** Chosen controllable factors (cotton/spandex core-spun yarn parameters) and their levels.

Factor notation	Factor name	Factors levels				
		1	2	3	4	5
*A*	Twist multiplier	4	4.2			
*B*	Spandex linear density (Denier)	22	33	44	78	
*C*	Spandex drafting ratio (%)	2.4	2.8	3.3	4	4.4

**Table 4:** Selected parameters and levels of a full 2 × 4 × 5 experimental design and their corresponding cotton/spandex core-spun yarn properties.

Run No	Controllable factors, their notations, coding and levels						Cotton/spandex core-spun yarn properties				
	***A***	***B***	***C***	***A***	***B***	***C***	Breaking load, cN	Breaking elongation, %	Breaking work, N.cm	Hairiness index	Total imperfection index
1	1	1	1	4	22	2.4	387.66	5.00	5.40	4.86	37.74
2	1	1	2	4	22	2.8	393.12	5.80	6.00	5.36	66.30
3	1	1	3	4	22	3.3	369.46	6.10	5.70	6.00	61.20
4	1	1	4	4	22	4	422.24	6.90	7.10	5.29	33.66
5	1	1	5	4	22	4.4	455.00	7.20	11.00	5.90	5.10
6	1	2	1	4	33	2.4	342.16	5.50	4.90	5.70	61.20
7	1	2	2	4	33	2.8	378.56	6.50	6.10	5.22	81.60
8	1	2	3	4	33	3.3	353.99	6.30	5.50	5.20	76.50
9	1	2	4	4	33	4	418.60	6.90	6.90	5.30	40.80
10	1	2	5	4	33	4.4	391.30	8.20	11.50	5.70	5.10
11	1	3	1	4	44	2.4	310.31	6.00	4.70	6.54	90.78
12	1	3	2	4	44	2.8	360.36	6.80	5.90	4.67	99.96
13	1	3	3	4	44	3.3	347.62	6.50	5.50	4.48	92.82
14	1	3	4	4	44	4	408.59	6.80	6.70	5.32	53.04
15	1	3	5	4	44	4.4	429.52	8.90	11.20	5.60	5.10
16	1	4	1	4	78	2.4	300.30	6.30	4.90	5.68	51.00
17	1	4	2	4	78	2.8	279.37	6.60	4.60	5.71	53.04
18	1	4	3	4	78	3.3	384.02	7.20	6.40	4.38	30.60
19	1	4	4	4	78	4	336.70	8.10	6.00	5.34	53.04
20	1	4	5	4	78	4.4	316.68	8.30	5.00	5.80	5.10
21	2	1	1	4.2	22	2.4	420.42	5.87	5.81	4.69	27.99
22	2	1	2	4.2	22	2.8	427.11	6.73	6.43	5.20	49.18
23	2	1	3	4.2	22	3.3	404.18	7.05	6.12	5.71	45.40
24	2	1	4	4.2	22	4	458.64	7.90	7.55	5.10	24.97
25	2	1	5	4.2	22	4.4	492.08	8.23	11.73	5.41	4.85
26	2	2	1	4.2	33	2.4	372.65	6.40	5.30	5.51	45.40
27	2	2	2	4.2	33	2.8	410.87	7.48	6.53	5.08	60.53
28	2	2	3	4.2	33	3.3	386.98	7.26	5.92	5.10	56.75
29	2	2	4	4.2	33	4	455.77	7.90	7.34	4.90	30.26
30	2	2	5	4.2	33	4.4	425.20	9.30	12.24	5.10	4.85
31	2	3	1	4.2	44	2.4	341.11	6.94	5.10	6.32	67.34
32	2	3	2	4.2	44	2.8	391.76	7.80	6.32	4.87	74.15
33	2	3	3	4.2	44	3.3	379.33	7.48	5.92	4.49	68.85
34	2	3	4	4.2	44	4	444.31	7.80	7.14	5.00	39.34
35	2	3	5	4.2	44	4.4	468.20	10.05	11.93	5.00	4.85
36	2	4	1	4.2	78	2.4	329.65	7.26	5.30	5.51	37.83
37	2	4	2	4.2	78	2.8	310.54	7.58	5.00	5.51	39.34
38	2	4	3	4.2	78	3.3	417.55	8.23	6.83	4.39	22.70
39	2	4	4	4.2	78	4	367.87	9.19	6.63	5.20	39.34
40	2	4	5	4.2	78	4.4	348.76	9.40	6.12	5.30	4.85

Depending on the different levels of the spandex monofilament and its corresponding drafting ratio, the amount of spandex in the final cotton/spandex core-spun yarns will differ. This variation, in turn, significantly affects the characteristic of this type of spun yarn as well as the performance characteristics of fabrics woven from it. The percentage of spandex present in the produced yarns at various spandex linear densities and drafting ratios was tabulated in Table 5.

**Table 5:** Spandex content (%) in cotton/spandex core-spun yarns at different combinations of drafting ratios and linear densities of spandex monofilament.

No	Spandex linear density, (denier)	Spandex linear density, (tex)	Spandex drafting ratio (%)	Percentage of spandex (%)
1	22	2.44	2.4	5.17
2	22	2.44	2.8	4.44
3	22	2.44	3.3	3.76
4	22	2.44	4	3.10
5	22	2.44	4.4	2.82
6	33	3.67	2.4	7.76
7	33	3.67	2.8	6.65
8	33	3.67	3.3	5.65
9	33	3.67	4	4.66
10	33	3.67	4.4	4.23
11	44	4.89	2.4	10.35
12	44	4.89	2.8	89.51
13	44	4.89	3.3	7.53
14	44	4.89	4	6.21
15	44	4.89	4.4	5.65
16	78	8.67	2.4	18.35
17	78	8.67	2.8	15.73
18	78	8.67	3.3	13.34
19	78	8.67	4	11.01
20	78	8.67	4.4	10.01
The linear density of yarn with spandex = 30/Ne (19.68tex)				

### Laboratory testing

Before testing, the forty cotton/spandex core-spun yarn samples were left in a standard and conditioned environment for 24 h at a temperature of 20 ± 2 °C and a relative humidity of 65% ± 2. All yarn properties were evaluated according to the appropriate ASTM standard test methods.

Using the USTER TENSORPID 5 measuring apparatus, the tensile characteristics of the cotton/spandex yarn samples were evaluated in terms of breaking work (N.cm), breaking elongation (%), and breaking load (cN) following the ASTM D2256 standard test procedure. Fifty individual measurements were recorded for each yarn type, and the average value was estimated. This means that the total tensile measurements for the undertaken yarn samples in this study were roughly two thousand. The hairiness index denotes the total length of all fibers protruding from the yarn surface per one centimeter of yarn length. Similarly, the imperfection index (IPI) of the yarn indicates the total number of neps, thick places, and thin places per one thousand meters of the measured yarn length. The thin places, thick places, and neps are characterized by the deviations in yarn diameter of -50%, + 50%, and + 200%, respectively, from the mean yarn diameter. It is noteworthy that both hairiness and imperfection indices were measured over one kilometer of the yarn length, repeated five times for each yarn sample. This means that each reading of the hairiness index of the yarn for each cycle represents the average of approximately 100,000 individual measurements. The hairiness and imperfection indices of the forty cotton/spandex yarn samples under study were examined using the USTER Tester 5 (Switzerland, model S400) following ASTM D5647 and ASTM D1425 standard test methods for hairiness index and imperfection index, respectively.

### Robust Taguchi design and its S/N ratios

To achieve excellent quality performance for any product without raising its price, optimizing all production process variables is a fundamental step in the robust Taguchi technique. It is worth noting that the Taguchi technique is primarily employed for the optimization of performance characteristics based on a single criterion^22^.

The realization of optimal parameters can be done using the signal-to-noise ratio (S/N ratio) for this modern approach.

Typically, there are three forms of S/N ratios, including the smaller-the-better, the higher-the-better, and nominal-the-best. Each mode can be measured using one of the following formulas^23^ :

- The smaller -the-better
  5 $$S/N = - 10 \times Log_{10} \left( {\frac{1}{n}\sum\limits_{i = 1}^{n} {y_{ij}^{2} } } \right)$$
- The higher–the–better
  6 $$S/N = - 10 \times Log_{10} \left( {\frac{1}{n}\sum\limits_{i = 1}^{n} {\frac{1}{{y_{ij}^{2} }}} } \right)$$
- The nominal – the best
  7 $$S/N = Log_{10} \left( {\sum\limits_{i = 1}^{n} {\frac{{\mathop y\limits^{ - 2} }}{{\mathop s\limits^{2} }}} } \right)$$

where y_ij_ represents the i^th^ result of the run at factor j, $\mathop y\limits^{\_}$ and, $\mathop S\limits^{2}$ refer to the mean and variance of the sample respectively, and n denotes the number of observations of the i^th^ experiment.

Furthermore, the arithmetic mean and standard deviation of any yarn characteristic can be evaluated by the following equations:

8 $$\mathop y\limits^{\_} = \frac{{y_{1} + y_{2} + y_{3} + ........... + y_{n} }}{n}$$

9 $$S^{2} = \frac{{\sum\limits_{i = 1}^{n} {\left( {y_{i} - \mathop y\limits^{\_} } \right)^{2} } }}{n - 1}$$

Since the objective of this study is to manufacture cotton/spandex composite yarn with high breaking load, high elongation at break, and high breaking work, as well as low hairiness and imperfection indices, it is therefore essential to maximize breaking load, breaking elongation, and breaking work, while minimizing hairiness and imperfection indices.

### Grey relational analysis (GRA)

The term “grey” points to the information that is incomplete, uncertain, or lacking full clarity. The Grey Relational Analysis (GRA) technique is an effective approach for addressing complex interactions between various performance parameters. Using this technique, the many performance criteria are converted into an equivalent single response characteristic, achieving simultaneous optimization. To execute this analysis, the following steps should be carried out sequentially: Normalization of S/N ratio data, calculation of the grey relational coefficients (GRCs), and the grey relational grade values (GRGs) are then determined.

Normalization is applied to reduce variability and to eliminate the impact of using different measuring units on the response characteristics, thereby enhancing the effectiveness of the subsequent analysis. Accordingly, the values of S/N ratios for each cotton/spandex composite yarn characteristic are normalized to fall between 0 and 1. Generally speaking, normalization is executed based on the kind of performance characteristic of the core-spun yarns, using a suitable equation. The following equations have been used for both the smaller-the-better and the-higher-the better^24^ .

10 $$y_{i}^{*} \left( k \right) = \frac{{y_{i}^{0} \left( k \right) - \min y_{i}^{0} \left( k \right)}}{{\max y_{i}^{0} \left( k \right) - \min y_{i}^{0} \left( k \right)}}\;{\text{For higher the better}}$$

11 $$y_{i}^{*} \left( k \right) = \frac{{\max y_{i}^{0} \left( k \right) - y_{i}^{0} \left( k \right)}}{{\max y_{i}^{0} \left( k \right) - \min y_{i}^{0} \left( k \right)}}\;{\text{For}}\;{\text{smaller}}\;{\text{the}} - {\text{better}}$$

where, $\max y_{i}^{0} \left( k \right)$ and $\min y_{i}^{0} \left( k \right)$ represent the biggest and smallest values of the original experimental data for the k^th^ response respectively, and the original value of the k^th^ response in the i^th^ experiment was denoted by $y_{i}^{0} \left( k \right)$. Typically, the highest normalized value which equal one is considered the superior performance for a given yarn property.

The normalized data were subsequently utilized to determine the grey relational coefficient (GRC), which quantifies the relationship between the actual and optimal normalized experimental data. The GRC values are typically determined by the following Equation. ^25^

12 $$\zeta_{i} (k) = \frac{{\Delta_{\min } + \zeta \Delta_{\max } }}{{\Delta_{oi} (k) + \zeta \Delta_{\max } }}$$

where Δ_oi_(k) denotes the deviation sequence between the comparability and reference sequences, which can be determined using the following well-known equation:

13 $$\Delta_{oi} (k) = \left\| {y_{0}^{*} (k) - y_{i}^{*} (k)} \right\|$$

where ζ denotes the distinguishing factor, which can take any values between one and zero. In this work, ζ was set as 0.5. The $y_{i}^{*} (k)$, and $y_{0}^{*} (k)$ represent the comparability and reference sequence respectively.

The average values of the grey relational grades can be determined by averaging the individual values of the grey relational coefficients using the following formula^26^ :

14 $$\mathop {\gamma_{j} }\limits^{\_} = \frac{1}{k}\sum\limits_{i = 1}^{m} {\left( {\gamma_{ij} } \right)}$$

where $\mathop {\gamma_{j} }\limits^{\_}$ represents the average value of the grey relational grade for the j^th^ experiment, and K denotes the number of the evaluated cotton/spandex core yarn properties.

It should be noted that the optimal sequence of corresponding parameters is obtained as the grey relational grade increases. Generally, the highest value of the grey relational grades is associated with the best multi-response characteristic. Thus, we can determine the optimum level of each controllable parameter and evaluate its effect using the average values of the grey relational grades^27,28^.

###  Analysis of variance

By examining the impact of individual core-spun yarn process variables, analysis of variance (ANOVA) is used to assess the significance of each controllable factor at 0.05 and 0.01 significance levels. Since the Taguchi technique can not separate the influence of a given factor, ANOVA makes up for it by computing each parameter’s percentage contribution to the total variation (total sum of squares)^29^.

Typically, the total sum of squares is divided into two sections: the total sum of squared deviation resulting from the controllable factors and that caused by the error. The F-values correspond to each control factor, defining its significance value, where a greater impact on the yarn performance property is associated with a greater F-value. In the ANOVA analysis, the p-value also identifies specifically the levels at which any factor impacts the yarn characteristic property^30,31^.

## Results and discussion

Before introducing the results and discussion, it is important to outline the hypothesis and expectations for this study: It was anticipated that the selected parameters, such as yarn twist multiplier, spandex liner density, and draft ratios, would significantly affect the cotton/spandex core-spun yarn properties at a statistically significant level. Also, the levels of the studied parameters were confined to those available in the Elmahla Elkobra company with the final produced product, i.e., woven fabric. Furthermore, the results of this study can be generalized only regarding the parameters under study; however, by investigating new parameters, surely the results will be changed.

To enhance the quality of cotton/spandex core-spun yarn, this study investigates various covering process parameters, as outlined in the aforementioned experimental section. Based on the aforementioned control factors—namely, twist multiplier, spandex monofilament linear density, and spandex monofilament drafting ratio—key yarn performance responses, including breaking load, breaking elongation, breaking work, hairiness index, and total imperfection index, were evaluated and optimized. To determine the optimal combination of control factors that provide the most desirable response variables, the Grey Relational Analysis (GRA) technique was employed.

In general, high yarn breaking load, breaking work, and elongation, along with low hairiness index and total imperfection index, are the best indicators of superior core-spun yarn performance. Accordingly, tensile properties are classified as 'the-higher-the-better’ criteria, while hairiness index and imperfection values are treated as 'the-lower-the-better’ criteria.

### Calculation of S/N ratios, grey relational coefficients, and grey relational grades

The signal-to-noise (S/N) ratio values of the cotton/spandex core-spun yarn covering process parameters, along with their corresponding normalized values, are presented in Table 6. These values were computed using Eqs. (5) through (7), based on the objectives of maximizing yarn tensile properties and minimizing hairiness and imperfection indices in the covering process.

**Table 6:** S/N ratios and their associated normalized values for the cotton/spandex core-spun yarn properties.

Runs	Breaking load		Breaking elongation		Breaking work		Hairiness Index		Imperfection Index	
	S/N ratio	Normalized S/N	S/N ratio	Normalized S/N	S/N ratio	Normalized S/N	S/N ratio	Normalized S/N	S/N ratio	Normalized S/N
1	51.769	0.579	13.979	0.000	14.648	0.164	-13.733	0.259	-31.536	0.678
2	51.891	0.603	15.269	0.213	15.563	0.271	-14.583	0.504	-36.430	0.864
3	51.351	0.494	15.707	0.285	15.118	0.219	-15.563	0.785	-35.735	0.838
4	52.511	0.730	16.777	0.462	17.025	0.444	-14.469	0.471	-30.542	0.640
5	53.160	0.862	17.147	0.523	20.828	0.891	-15.417	0.743	-14.151	0.017
6	50.685	0.358	14.807	0.137	13.804	0.065	-15.118	0.657	-35.735	0.838
7	51.563	0.537	16.258	0.376	15.707	0.288	-14.353	0.438	-38.234	0.933
8	50.980	0.418	15.987	0.331	14.807	0.183	-14.320	0.428	-37.673	0.912
9	52.436	0.714	16.777	0.462	16.777	0.414	-14.486	0.476	-32.213	0.704
10	51.850	0.595	18.276	0.709	21.214	0.936	-15.118	0.657	-14.151	0.017
11	49.836	0.186	15.563	0.261	13.442	0.022	-16.312	1.000	-39.160	0.968
12	51.135	0.450	16.650	0.441	15.417	0.254	-13.386	0.160	-39.997	1.000
13	50.822	0.386	16.258	0.376	14.807	0.183	-13.026	0.056	-39.353	0.976
14	52.226	0.672	16.650	0.441	16.522	0.384	-14.518	0.485	-34.492	0.791
15	52.660	0.760	18.988	0.826	20.984	0.909	-14.964	0.613	-14.151	0.017
16	49.551	0.128	15.987	0.331	13.804	0.065	-15.087	0.648	-34.151	0.778
17	48.924	0.000	16.391	0.398	13.255	0.000	-15.133	0.661	-34.492	0.791
18	51.687	0.562	17.147	0.523	16.124	0.337	-12.830	0.000	-29.714	0.609
19	50.545	0.330	18.170	0.691	15.563	0.271	-14.551	0.494	-34.492	0.791
20	50.012	0.221	18.382	0.726	13.979	0.085	-15.269	0.700	-14.151	0.017
21	52.474	0.722	15.371	0.230	15.290	0.239	-13.427	0.172	-28.941	0.579
22	52.611	0.750	16.555	0.425	16.159	0.342	-14.323	0.429	-33.836	0.766
23	52.131	0.652	16.960	0.492	15.735	0.292	-15.136	0.662	-33.140	0.739
24	53.229	0.876	17.957	0.656	17.557	0.506	-14.151	0.380	-27.948	0.542
25	53.841	1.000	18.303	0.713	21.386	0.957	-14.658	0.525	-13.715	0.000
26	51.426	0.509	16.130	0.355	14.492	0.146	-14.820	0.572	-33.140	0.739
27	52.274	0.681	17.473	0.576	16.296	0.358	-14.117	0.370	-35.639	0.834
28	51.754	0.576	17.220	0.535	15.441	0.257	-14.151	0.380	-35.079	0.813
29	53.175	0.865	17.957	0.656	17.319	0.478	-13.797	0.278	-29.619	0.605
30	52.572	0.742	19.366	0.889	21.756	1.000	-14.151	0.380	-13.715	0.000
31	50.658	0.353	16.827	0.470	14.151	0.105	-16.020	0.916	-36.565	0.869
32	51.860	0.597	17.838	0.637	16.020	0.325	-13.742	0.262	-37.402	0.901
33	51.580	0.540	17.473	0.576	15.441	0.257	-13.041	0.061	-36.758	0.877
34	52.954	0.820	17.838	0.637	17.074	0.449	-13.976	0.329	-31.897	0.692
35	53.409	0.912	20.040	1.000	21.536	0.974	-13.976	0.329	-13.715	0.000
36	50.361	0.292	17.220	0.535	14.492	0.146	-14.820	0.572	-31.557	0.679
37	49.842	0.187	17.596	0.597	13.976	0.085	-14.820	0.572	-31.897	0.692
38	52.414	0.710	18.303	0.713	16.694	0.404	-12.841	0.003	-27.120	0.510
39	51.314	0.486	19.266	0.872	16.430	0.374	-14.323	0.429	-31.897	0.692
40	50.850	0.392	19.466	0.905	15.735	0.292	-14.492	0.477	-13.715	0.000

Using Eqs. (10) through (11), the grey relational coefficients were computed based on the normalized values of S/N ratios following the standard steps of the grey relational analysis technique. In the estimation of the Grey Relational Coefficients (GRCs), the distinguishing coefficient was set to a value of 0.5. Following the calculation of the GRCs, the corresponding Grey Relational Grades (GRGs) were determined using Eq. (14).

**Fig. 2:**
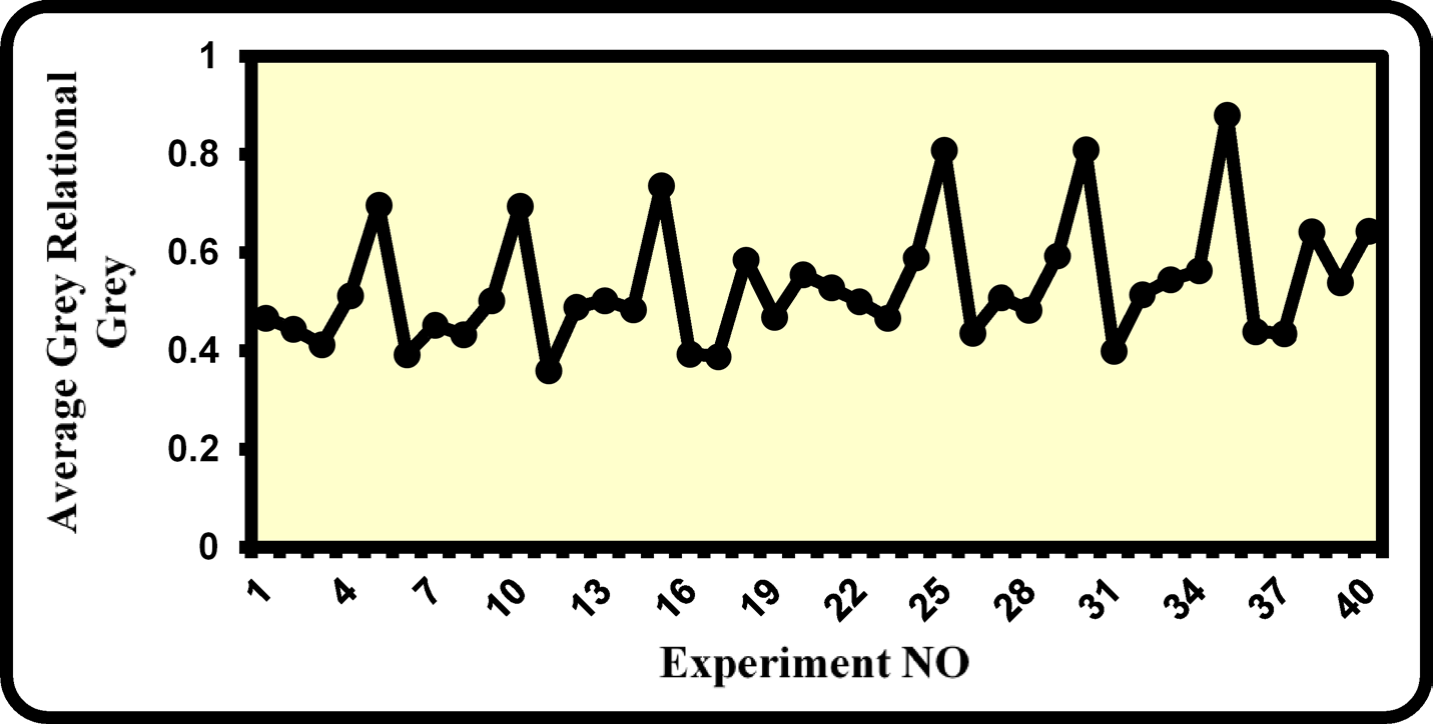
Relationship between experiment numbers and the values of the grey relational grades.

The grey relational coefficients (GRCs) and their corresponding grey relational grades (GRGs) and resulting ranks were introduced in Table 7. In general, the optimum multi-response characteristic corresponds to the highest grey relational grade value. Figure 2 depicts the forty conducted experiments and their associated grey relational grades.

**Table 7:** Grey relational coefficients (GRCs) and their associated grade values (GRGs) for cotton/polyester core-spun yarn properties.

Run No	Grey relational coefficients (GRC)					Grey Relational grade (GRG)	Rank
	Breaking load	Breaking elongation	Breaking work	Hairiness index	Total imperfection index		
1	0.543	0.333	0.374	0.658	0.424	0.467	28
2	0.558	0.388	0.407	0.498	0.366	0.444	30
3	0.497	0.412	0.390	0.389	0.374	0.412	35
4	0.649	0.482	0.473	0.515	0.438	0.511	18
5	0.783	0.512	0.821	0.402	0.968	0.697	5
6	0.438	0.367	0.348	0.432	0.374	0.392	38
7	0.519	0.445	0.413	0.533	0.349	0.452	29
8	0.462	0.428	0.380	0.539	0.354	0.432	34
9	0.636	0.482	0.461	0.513	0.415	0.501	21
10	0.553	0.632	0.887	0.432	0.968	0.694	6
11	0.380	0.404	0.338	0.333	0.341	0.359	40
12	0.476	0.472	0.401	0.758	0.333	0.488	23
13	0.449	0.445	0.380	0.899	0.339	0.502	20
14	0.604	0.472	0.448	0.508	0.387	0.484	24
15	0.675	0.742	0.846	0.449	0.968	0.736	4
16	0.364	0.428	0.348	0.435	0.391	0.393	37
17	0.333	0.454	0.333	0.430	0.387	0.388	39
18	0.533	0.512	0.430	1.000	0.451	0.585	11
19	0.427	0.618	0.407	0.503	0.387	0.469	26
20	0.391	0.646	0.353	0.417	0.968	0.555	13
21	0.643	0.394	0.397	0.744	0.463	0.528	16
22	0.667	0.465	0.432	0.538	0.395	0.499	22
23	0.590	0.496	0.414	0.430	0.404	0.467	27
24	0.801	0.593	0.503	0.568	0.480	0.589	10
25	1.000	0.636	0.920	0.488	1.000	0.809	3
26	0.504	0.437	0.369	0.467	0.404	0.436	32
27	0.611	0.541	0.438	0.575	0.375	0.508	19
28	0.541	0.518	0.402	0.568	0.381	0.482	25
29	0.787	0.593	0.489	0.643	0.452	0.593	9
30	0.660	0.818	1.000	0.568	1.000	0.809	2
31	0.436	0.485	0.359	0.353	0.365	0.400	36
32	0.554	0.579	0.426	0.656	0.357	0.514	17
33	0.521	0.541	0.402	0.892	0.363	0.544	14
34	0.735	0.579	0.476	0.603	0.420	0.562	12
35	0.850	1.000	0.951	0.603	1.000	0.881	1
36	0.414	0.518	0.369	0.467	0.424	0.438	31
37	0.381	0.554	0.353	0.467	0.420	0.435	33
38	0.633	0.636	0.456	0.993	0.495	0.643	8
39	0.493	0.796	0.444	0.538	0.420	0.538	15
40	0.451	0.841	0.414	0.512	1.000	0.643	7

From Table 7 and Fig. 2, it is evident that the greatest value of the grey relational grade is accompanied by cotton/spandex core spun yarn made with the parameters outlined in Experiment No. 35. Consequently, among 40 practical experiments, Experiment No. 35 produced the best multi-performance characteristics. It is also noticed that Experiment No. 11 has the lowest grey relational grade value among all experiments. Table 7 demonstrates the average grey relational grade values for each of the 40 experiments. It should be noted that the association degree between comparative and referential sequences is typically represented by the grey relational grade. The grey relational grade value increases when the relationship between the two sequences becomes stronger. Therefore, the optimal outcome for this study is the highest value of the grey relational grade, which also yields the highest breaking load, largest breaking elongation, and breaking work, and minimal hairiness index and total imperfection index. According to Table 8, it can be noticed that the best grey relational grade values for the different undertaken control factors, namely A, B, and C, correspond to levels A_2_, B_3_, and C_5_. Consequently, A_2_B_3_C_5_ represents the optimum combination of cotton/spandex core-spun-yarn covering parameters to maximize breaking load, breaking elongation, breaking work, and simultaneously minimize total imperfection and hairiness indices. Additionally, it can be proved that the factor with the highest gain value has the most influence on the performance characteristics. Table 8 also demonstrates that the spandex drafting ratio and twist multiplier have the greatest influence, while spandex linear density has the lowest impact on the performance characteristics of the cotton/spandex core-spun yarns.

**Table 8:** Mean Grey relational grade values at different levels of control factors.

Control factors	Levels					Gain (Max–Min)
	1	2	3	4	5	
Twist multiplier	0.498	0.566^a^				0.068
Spandex linear density	0.542	0.529	0.547^a^	0.509		0.038
Spandex drafting ratio	0.427	0.466	0.508	0.531	0.728^a^	0.301
**(**Total mean grey relational grade = 0.532 – a refers to the optimal level**)**						

**Fig. 3:**
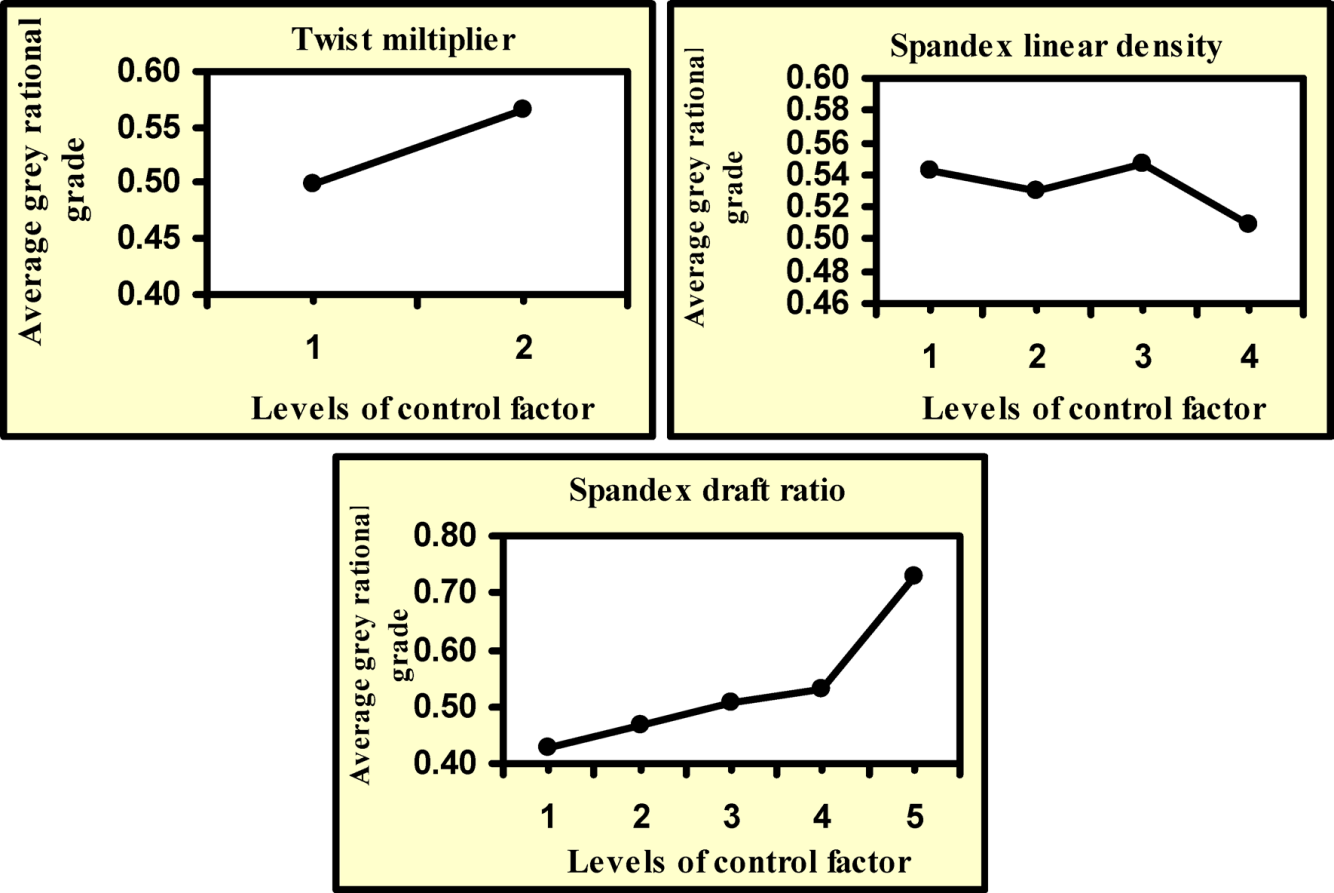
Average values of grey relational grades at different levels of the control factors.

Figure 3 depicts how each control factor influences the average values of the grey relational grades. From this figure, it is clear that the optimum levels to maximize cotton/spandex core-spun yarn tensile characteristics while minimizing its imperfection and hairiness indices are the third level of spandex linear density, the fifth level of the spandex monofilament drafting ratio, and the second level of the core-spun yarn twist multiplier. From this figure and the statistical analysis, it is noticed that the average value of the grey relational grade is positively influenced by both the twist multiplier and the drafting ratio, while it is negatively impacted by the spandex linear density. It was estimated that increasing the core-spun yarn twist multiplier from 4 to 4.2 leads to an increase in the average grade value by about 13.7%; on the other hand, the grey relational grade increases by around 70% when the drafting ratio of the spandex monofilament increases. In contrast, increasing the linear density of spandex monofilament was found to reduce the average value of the grey relational grade by around 6.2%.

### The analysis of variance (ANOVA) results

The impact of each control factor on the average values of the grey relational grades was evaluated using the analysis of variance (ANOVA), and the corresponding contribution of each factor was also tabulated in Table 9. It is noticed from this table that all twist multipliers and drafting ratios of cotton/spandex core spun yarn have a profound impact on the grey relational grades at the 0.01 significance level, while linear density of the core-spun yarn was found to have no influence on the average value of the grey relational grades. It is also noticed that the spandex monofilament drafting ratio has the highest influential impact on the multiple performance characteristics, followed by the twist multiplier and the influence of spandex linear density, respectively. The statistical analysis revealed that the spandex monofilament drafting ratio and twist multiplier were found to be responsible for 67% and 24% of the impacts on the grey relational grades, respectively, while the spandex linear density was evaluated to contribute by about 9% to the effect. This suggests that the spandex monofilament drafting ratio impacts the cotton/spandex core-spun yarn characteristics more than the other control factors. Additionally, it is evident that the values of significance levels (p-values) and F-values listed in Table 9 confirm the percentage contribution of each control factor in the effect on the response characteristics. As the contribution percentage of the effect of control factors on the multi-response criteria increases, the F-value increases, and hence the P-values decrease. It also observed that gain values shown in Table 8 agree with the contribution percentage of each control factor.

**Table 9:** Results of Analysis of variance (ANOVA) for the grey relational grades.

Source of variation	DF	SS	MS	F	P-value	Contribution %
Twist multiplier	1	0.046	0.046	12.690	0.001	24
Spandex linear density	3	0.009	0.003	0.810	0.499	9
Spandex drafting ratio	4	0.436	0.109	30.060	0.000	67
Error	31	0.112	0.004			
Total	39	0.603				

### Confirmation test

Following the identification of the best control parameters for the process, the confirmation test is conducted to confirm the improvements in the performance of the cotton/spandex core-spun yarn. The confirmation test incorporates the optimum levels of the control parameters. The following equation can be utilized to predict the grey relational analysis.

15 $$\gamma_{pred} = \gamma_{t} + \sum {\left( {\gamma_{i} - \gamma_{t} } \right)}$$

where γ_t_ denotes the overall average of grey relational grade values, γ_i_ represents the average of grey relational grade at optimal levels as determined by Table 8.

From the above equation, it was determined that the predicted GRG value is approximately 0.78. The closeness of both predicted and experimental values of GRG to each other is referred to as the prediction of the percentage error. It was estimated that the percentage of the predicted error is around 11%. The grey relational grade was enhanced by about 89% over the initial conditions. Table 10 introduced the grey relational grades’ initial, predicted, and optimum values.

**Table 10:** Results of predicted, experimental, and initial grey relational grades for confirmation experiment.

	Initial conditions	Optima conditions	
		Predicted	Experimental
Levels	A_1_B_1_C_1_	A_2_B_3_C_5_	A_2_B_3_C_5_
Breaking load, cN	387	………	468
Breaking elongation, %	5	………	10.01
Breaking work, N.cm	5.4	………	11.9
Hairiness index	4.86	………	4.9
Total imperfection index	38	………	5
GRG	0.467	0.78	0.88

## Conclusion

In this study, the influences of three controllable factors, namely yarn twist multiplier, spandex monofilament linear density, and spandex drafting ratio, on five key performance parameters of cotton/spandex core-spun yarns, including yarn breaking load, breaking elongation, breaking work, hairiness, and imperfection indices, were investigated. The primary objective was to optimize the performance of core-spun yarns at different levels of these control factors.

Multi-objective optimization was employed using grey relational analysis in conjunction with the Taguchi technique from a 2 × 4 × 5 full factorial design with three factors with two, four, and five different levels, respectively. Forty cotton/spandex core-spun yarns were produced throughout this study. Analysis of variance was used to assess the significance of controllable factors on the cotton/spandex characteristics. The findings of this study can be summed up as follows:

- The average values of the grey relational grades revealed that the best parameter combination was estimated as A_2_ B_3_ C_5_, i.e., a 4.2 twist multiplier, 44 denier, and a 4.4 drafting ratio of spandex monofilament.
- The correlation between the experimental and predicted values of grey relational values was found to be very good.
- For improving the cotton/spandex core-spun yarns, the drafting ratio of the spandex monofilament was found to be the most influential parameter, followed by the net value of the yarn linear density with 67% and 24% of contribution, respectively.
- Linear density of the spandex monofilament has the least contribution to the performance characteristics of the cotton/spandex core yarn.
- Compared to the initial conditions, the cotton/spandex core-spun performance characteristics were greatly enhanced by about 89% using the optimization technique.

Finally, it is worth noting that this study can benefit producers, industrialists, and spinners by enabling them to adjust their spinning machines using scientific and mathematical approaches, which leads to reduced processing faults, product defects, and improved product quality, ultimately increasing company profitability.

## Data Availability

The data that support the findings of this study are available on reasonable request from the principal author.
